# Evaluation of Potential Toxic Elements in Soils from Three Urban Areas Surrounding a Steel Industrial Zone

**DOI:** 10.3390/toxics13050351

**Published:** 2025-04-28

**Authors:** Georgios Charvalas, Aikaterini Molla, Alexios Lolas, Elpiniki Skoufogianni, Savvas Papadopoulos, Evaggelia Chatzikirou, Christina Emmanouil, Olga Christopoulou

**Affiliations:** 1 Department of Planning and Regional Development, University of Thessaly, 38334 Volos, Greece; gcharval@gmail.com (G.C.); ochris@uth.gr (O.C.); 2 Hellenic Republic, Ministry of Rural Development and Food, 11523 Athens, Greece; amolla@uth.gr; 3 Laboratory of Marine Biology, Department of Ichthyology & Aquatic Environment, University of Thessaly, Fytokou Street, 38446 Volos, Greece; allolas@uth.gr; 4 Laboratory of Agronomy and Applied Crop Physiology, Department of Agriculture, Crop Production and Rural Environment, University of Thessaly, 38446 Volos, Greece; 5 Department of Agrotechnology, University of Thessaly, 41110 Larissa, Greece; psavvas1@yahoo.gr; 6 Institute of Industrial and Forage Crops, Hellenic Agricultural Organization—DEMETER, 41335 Larissa, Greece; evaggelia1966@yahoo.gr; 7 School of Spatial Planning and Development, Aristotle University of Thessaloniki, 54124 Thessaloniki, Greece; chemmanouil@plandevel.auth.gr

**Keywords:** PTEs, GIS, soil contamination, contamination factor, pollution load index, geo-accumulation index, ICP-MS, Volos, Greece

## Abstract

The urban zone around the city of Volos, a Greek city with a historically industrialized profile, faces threats arising from Potential Toxic Element (PTE) contamination. The scope of this study is to determine the contamination levels of 10 PTEs in three urban areas which are located near the industrial zone in the city of Volos. For this purpose, a total of 30 soil samples from parks, playgrounds and roadsides were collected from the Agios Georgios, Velestino and Rizomilos areas (Magnesia, Central Greece). The sampling was conducted in June 2022 and the concentrations of chromium (Cr), nickel (Ni), copper (Cu), arsenic (As), cadmium (Cd), lead (Pb), iron (Fe), manganese (Mn), cobalt (Co) and zinc (Zn) were measured through inductively coupled plasma mass spectrometry (ICP-MS). The Contamination Factor (CF), Pollution Load Index (PLI) and Geo-accumulation Index (Igeo) revealed moderate pollution in most cases, whereas in some sites the contamination was significant for Ni or for As. Principal Component Analysis showed concomitant changes for some PTEs in Component 1 and for others in Component 2, explaining approximately 67% of the variation. K-means Cluster Analysis showed two distinct groups of PTE-impacted sites within these urban areas. It can be postulated that industrial activities may have a carry-over effect on the soil in residential areas. Frequent monitoring of areas deemed as “contaminated” and time-series data are needed to examine in depth the soil pollution in cities and its possible shifts in relation to the changes in industrialization status in the extended urban areas.

## 1. Introduction

Soil is among the most complex biomaterials on Earth and plays a crucial role in human life and health [1]. It impacts food availability and quality, acts as a natural filter for water, detoxifies pollutants and facilitates human exposure to various chemical minerals [2]. Furthermore, soil serves as a habitat for numerous organisms that are essential for nutrient cycling and supporting a healthy environment for humans. Soil affects human health directly and indirectly, and the impacts of soil on health can be beneficial or adverse [1].

Rapid urbanization and industrialization have significantly contributed to the degradation of urban environments. Urban surface soil, as a key component of urban ecosystems, acts as the main repository for various pollutants, including PTEs [2,3,4].

PTEs comprise elements with a density greater than 5 g cm^−3^ and non-metals, such as arsenic (As) [5]. Some PTEs are persistent in the environment and can pose significant toxicity risks to plants, animals and humans [6]. PTEs are non-degradable, harmful to all forms of life and can cause serious health-related adverse effects.

Soil contamination by Potential Toxic Elements (PTEs) in urban industrial areas is a major threat, as these pollutants can easily enter the food chain and pose serious health problems to plants, animals and humans.

The presence of PTEs in soils may originate from both natural and anthropogenic sources [7,8]. The anthropogenic inputs of PTEs include agricultural practices, mining operations, waste disposal and industrial activities [9].

The steel industry is known to be a significant source of PTE contamination through the emission of pollutants in the form of dust and gases [10]. Although steel production plays a significant role in the economy, it is also a major source of environmental pollution. This process generates large amounts of dust, which can remain suspended in the air and can be dispersed over vast areas by wind and rain, eventually accumulating in soils [11]. Solid and liquid wastes, which are produced by steel plants and their materials, often contain significant levels of PTEs such as As, Cd, Co, Cr, Cu, Fe, Mn, Ni, Pb and Zn, which are potentially harmful [12,13]. 

The threat of contamination by PTEs in urban soils located close to steel industrial plants poses a significant worldwide concern. These soils are exposed to higher levels of pollutants due to emissions and waste from the steel manufacturing processes. Over time, PTEs can accumulate in the soil, increasing the risk of their transfer to local water sources, crops and eventually the food chain. This situation not only compromises soil quality and ecosystem health [8] but also poses direct risks to the residents living in nearby areas. Human exposure to PTEs can lead to medical problems through dermal contact, ingestion and inhalation [6,14,15]. Inhalation of contaminated soil can lead to severe respiratory health problems, including coccidioidomycosis, acute bronchial inflammation, chronic bronchitis, emphysema and fibrotic changes to the lungs [16,17]. Soil ingestion can happen either intentionally, as in the case of geophagy, or unintentionally, through hand-to-mouth contact. This practice is very common among children [18,19].

Globally, previous studies have reported the contamination of PTEs in urban soils for various cities surrounded by steel industries. Lage et al. [20] demonstrated that the surface soils are influenced by airborne elements from both natural and anthropogenic sources and revealed that the levels of some elements were very high in soils near a heavily industrialized area in Northern Spain. Furthermore, significant contamination of PTEs has been detected in soils in public playgrounds and urban parks [21,22,23].

This contamination status can be effectively communicated with the use of pollution indices. The most widely used indices that assess PTE soil contamination are CF, PLI and Igeo [24].

A highly industrialized city in mainland Greece (Volos) is situated in the Magnesia Prefectural Unit. In the area around the city of Volos, the capital of Magnesia, several industries existed, such as steel and cement factories. Established in 1963, the steel plant in Volos city is one of the largest steel production sites, not only in Greece but also in the Balkans.

Hence, the aim of the present study was to monitor the levels of PTEs in the soils of parks, playgrounds and roadsides in three urban areas located near the steel plant and the second industrial zone of the city of Volos (Central Greece) and to explore the interrelations between the PTEs. These results are important because (a) research on PTE pollution in recreational urban areas is limited [4] and (b) the pollution effects of heavy industries are mostly focused on nearby agricultural land, while the urban aspect is underrepresented. Furthermore, this research may aid in creating an up-to-date database on inorganic soil pollution for Greece and the south of the Mediterranean in general and in unraveling the intricacies between pollution sources and soil contamination.

## 2. Materials and Methods

### 2.1. Study Area

**Figure 1:**
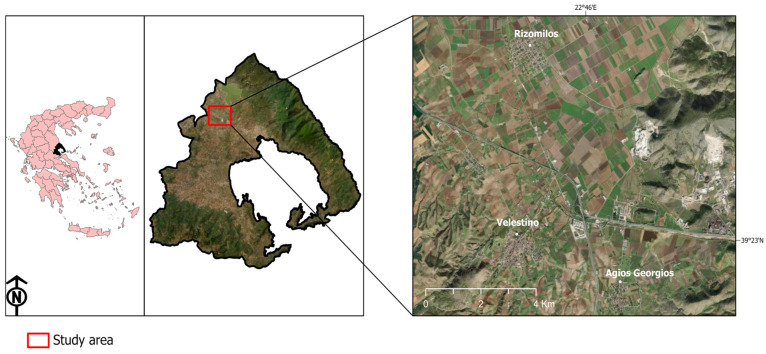
The Prefecture of Magnesia in Central Greece and the studied urban areas.

Volos is a coastal city in Thessaly Prefecture and the Magnesia Prefectural Unit (Central Greece), with a population of around 150,000 inhabitants. The whole area is characterized by a Mediterranean climate with hot, dry summers and cool, humid winters. The second industrial zone of the city is located 12–14 km west of Volos, where a steel plant is also situated. The research was carried out in three urban areas around the steel plant, named Agios Georgios (AG), Velestino (VL) and Rizomilos (RIZ) (Figure 1). These areas are within 6.5, 7.0 and 10 km from the steel industry, respectively.

### 2.2. Sampling and Chemical Analysis

**Figure 2:**
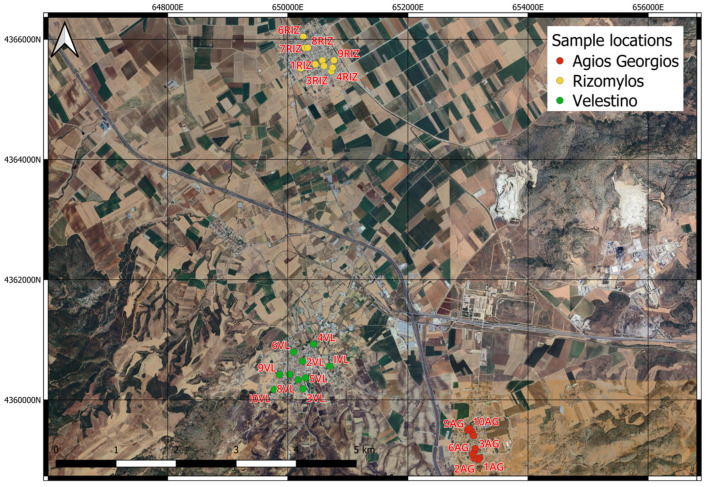
Location map of the samples collected from the three urban areas.

A total of thirty surface samples (at a depth of 0–10 cm) were collected from the three studied surrounding areas of the steel industry, which is located in the Magnesia Prefectural Unit. The soil samples (10 samples of each area) were collected from parks, playgrounds and roadsides from the studied urban areas in June 2022 (Figure 2).

Each soil sample, consisting of three different sub-samples, was collected from a 1 m × 1 m square plot and thoroughly mixed to create a composite sample. The soil samples were then air-dried, followed by drying in a forced-draught oven (L101-0AB—Chemist EU, Niel, Belgium) at 60 °C until a constant weight was achieved. Finally, they were sieved through a 63 μm mesh and placed in plastic containers. Ten PTEs were analyzed per sample: chromium (Cr), nickel (Ni), copper (Cu), arsenic (As), cadmium (Cd), lead (Pb), iron (Fe), manganese (Mn), cobalt (Co) and zinc (Zn). All the reagents used for the digestion of samples were of analytical grade. Ultrapure nitric acid (HNO_3_, 65% *w*/*w*, Chemlab, Zedelgem, Belgium) and supra-pure hydrogen peroxide (H_2_O_2_, 30% *w*/*w*, Chemlab, Zedelgem, Belgium) were utilized for the digestion. The digestion procedure lasted for a total of five days. On the first day, 1 g of sample (precisely recorded) was weighed and placed in a 50 mL Erlenmeyer flask. Subsequently, 5 mL of HNO_3_ was added, and the samples were left undisturbed (without combustion) for 24 h. On the second day, the samples were heated in a boiling sand bath at a temperature of 85–95 °C for 4–5 h, with the temperature being recorded. On the third day, an additional 5 mL of HNO_3_ was added, followed by combustion at 85–98 °C for another 4–5 h. On the fourth and fifth days, 3 mL of H_2_O_2_ was added each day, with subsequent heating at 85–98 °C for 4–5 h. On the last day, each sample was filtered into a 50 mL volumetric flask. Three replicates were conducted for each sample. The pseudo-total concentration of the digestate was measured using inductively coupled plasma mass spectrometry (Agilent 7700 Series ICP-MS—Conquer Scientific, Poway, CA, USA) [25]. Operating conditions of the method are given in Table S1. Τhe analysis was performed on the KED (kinetic energy discrimination) mode by using He as the collision gas. This method was chosen as it can be applied to almost all interferences using a single set of operating conditions and does not require an in-depth knowledge of the sample prior to analysis. Reference unpolluted soil (QCS) routinely used in the laboratory was spiked with known concentrations of the elements (LGC Standards, Teddington, UK), and the recovery rate was the following: Cr (94.2–104.9%), Mn (99.3–112.8%), Fe (97.8–97.9%), Co (102.7–113.4%), Ni (96.9–105.8%), Cu (90.0–97.4%), Zn (85.3–94.2%), As (99.2–99.7%), Cd (95.7–100.4%) and Pb (82.1–88.6%). The limit of detection and limit of quantitation of each element are given in Table S2. Soil pH (1:2.5 distilled H_2_O) was measured with a pH meter (Consort C5020 Multi-Parameter Analyzer-Fisher Scientific, Porto Salvo, Spain) [26,27,28,29].

The coordinates of each sample position were recorded using the Global Positioning System (GPS), as shown in Table S3.

### 2.3. Soil Contamination Assessment Indices

The soil contamination assessment was conducted using the 3 following parameters, namely, CF, PLI and Igeo [30,31].

#### 2.3.1. Contamination Factor (CF)

The CF was calculated to determine the PTE with the greatest environmental impact, and it was measured using the formula proposed by Hakanson [32]:

CF = C_sample_/C_Background_ (1)

where C_sample_ is the concentration of the PTE in the soil and C_Background_ is the concentration of the PTE in the background (mg kg^−1^, the values derived from Kabatas-Pendias [33] and were for Cr = 59.5, Mn = 480, Co = 11.3, Ni = 29, Cu = 38.9, Zn = 70, As = 6.83, Cd = 0.41 and Pb = 27).

#### 2.3.2. Pollution Load Index (PLI)

The PLI, a quick tool that compares the cumulative pollution status between different sites, was calculated using the equation given by Tomlinson et al. [34,35]:

PLI = (CF_1_ X CF_2_ X………X CF_n_)^1/n^ (2)

where CF is the Contamination Factor, CF_n_ is the Contamination Factor for the nth element and n is the number of the studied PTEs. The indices of the PLI are detailed in Table S4.

#### 2.3.3. Geo-Accumulation Index (Igeo)

The Geo-accumulation Index was applied to assess the level of PTE contamination in the soil samples. This index was calculated using Müller’s equation [36].

Igeo = log_2_(C/1.5 C_B_) (3)

where C is the concentration of the PTE in the soil samples and C_B_ is the concentration of the PTE in the background (the background values derived from Kabatas-Pendias) [33]. A factor of 1.5 was introduced to account for potential variations in the background values caused by lithogenic factors. The Igeo can be categorized into seven grades. The grades are presented in Table S5.

### 2.4. Geochemical Mapping

Maps that show the degree of pollution for each PTE were generated by using Geographical Information System (QGIS version 3.34.14) software.

### 2.5. Statistical Analysis

Differences between PTE concentrations for the three areas (AG, VL and RIZ) were assessed through a one-way ANOVA followed, whenever significant, by a Tukey HSD test with a level of significance of 95% (*p* < 0.05). Whenever homogeneity of variances was not satisfied, Welch’s ANOVA was utilized instead.

The PTE distribution within the 30 samples as well as the pH variation were subjected to a Principal Component Analysis (PCA) to describe the system with a new set of reduced variables [37]. Prior to performing the PCA, the suitability of the data for factor analysis was assessed. A hierarchical cluster analysis on the z-scores of the aforementioned parameters based on Ward’s method was performed. This was followed by a K-means Cluster Analysis that allocated all the examined cases into two clusters with different characteristics each [38]. All analyses were performed using SPSS 26.0 (IBM, Armonk, NY, USA).

## 3. Results

### 3.1. Assessment of Soil Sample Parameters

The soil pH ranged from 7.99 to 8.33 in AG, from 7.86 to 8.32 in VL and from 7.98 to 8.39 in RIZ. Generally, all the samples were alkaline (see Table S6).

### 3.2. Potential Toxic Element Concentration in Sampled Soils

**Figure 3:**
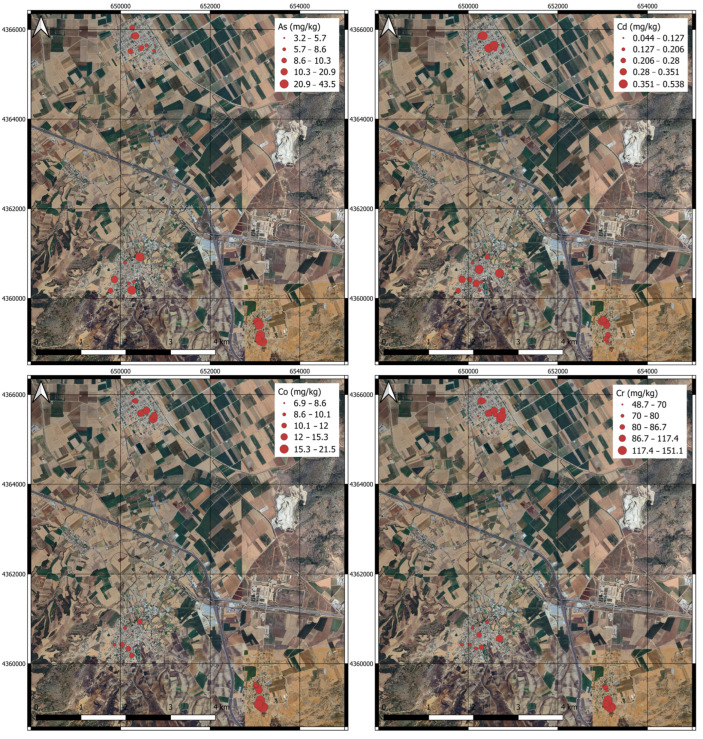

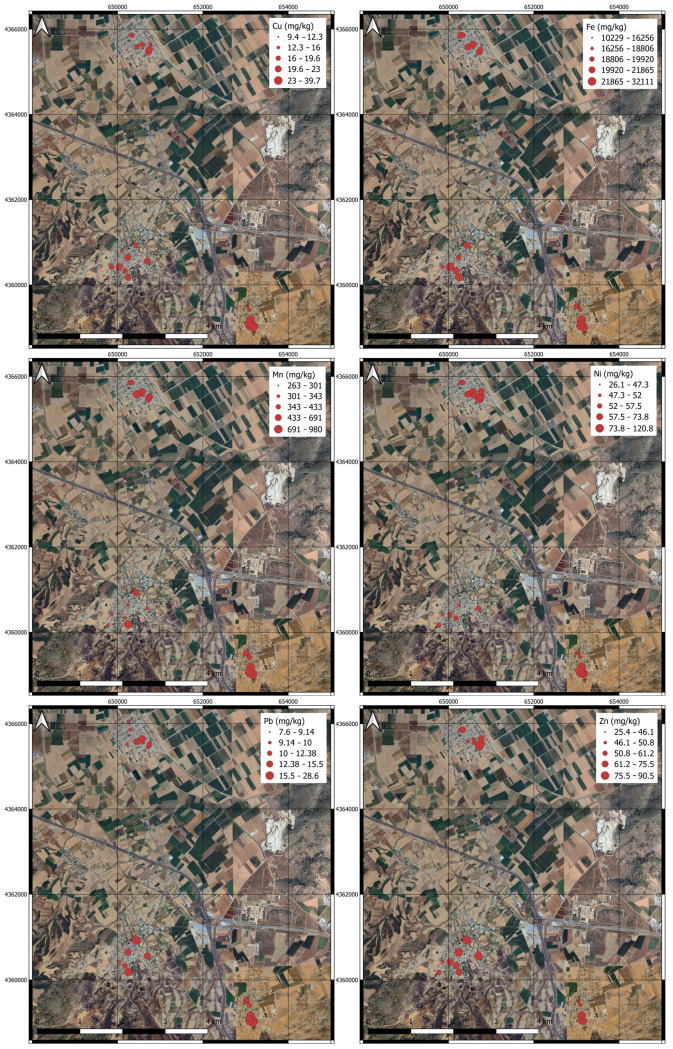
Geochemical maps of the studied PTEs in three urban areas.

Pseudo-total concentrations of the PTEs were measured in the soil samples as previously described. The results are presented in Table S6. Among the PTEs, the highest mean value was noticed in iron (Fe), which ranged from 17,236.2 to 26,123.4 mg kg^−1^ in AG, from 10,229.2 to 25,780.7 mg kg^−1^ in VL and from 1276.0 to 21,598.2 mg kg^−1^ in RIZ. The highest mean contents of chromium (Cr) (102.1 mg kg^−1^), manganese (Mn) (610.3 mg kg^−1^), iron (Fe) (21,797.7 mg kg^−1^), cobalt (Co) (15.4 mg kg^−1^), nickel (Ni) (66.8 mg kg^−1^), copper (Cu) (24.1 mg kg^−1^), arsenic (As) (19.7 mg kg^−1^) and lead (Pb) (14.0 mg kg^−1^) were measured in the AG urban area, and the contents of zinc (Zn) (62.6 mg kg^−1^) and cadmium (Cd) (0.276 mg kg^−1^) in VL (Figure 3).

These values were compared to set limits, whenever a relevant limit could be found, and, more specifically, to (a) the Greek national limits for soil that can receive sewage sludge as soil [39] amendment; (b) the Canadian Soil Quality Guidelines (SQGs) for urban soils [40]; and (c) the Dutch Target Values [41]. The values were also compared to global background levels according to [33] or [42]. These comparisons are shown in Table 1.

It is evident that the majority of the examined soils show high values of PTEs when compared to the background levels of Kabata-Pendias [33]. This is most prominent for Cr (93.3% of the samples) and for Ni (93.3% of the samples), as well as for As (63.3%). Even though not entirely relevant for an urban setting, the concentrations were compared to the maximum soil limits (for alkaline pH) for the use of sewage sludge as a fertilizer. These limits were set quite recently for Greece and are significantly lower than those in the corresponding EU Directive [43]. In this case, there was a limit exceedance in the samples for Cr (26.6%), Ni (2 samples) and As (2 samples). The Canadian SQGs have been developed to protect aquatic and terrestrial ecosystems, as well as human health, and they differentiate between scenarios such as residential areas, commercial areas and industrial settings. When compared to the limits for the residential areas, there were exceedances in many cases, especially for Ni and Cr. When compared to the Dutch Target Values, which supposedly represent maximum safe concentrations, there was an exceedance for Ni (86.6%), Cr (26.6%) and Co (46.6%), as well as in four samples for As. These exceedances raise concerns since humans may encounter these PTEs through various routes, and this may lead to unacceptable carcinogenic or non-carcinogenic risks for residents (adults or children) [44,45,46].

**Figure 4:**
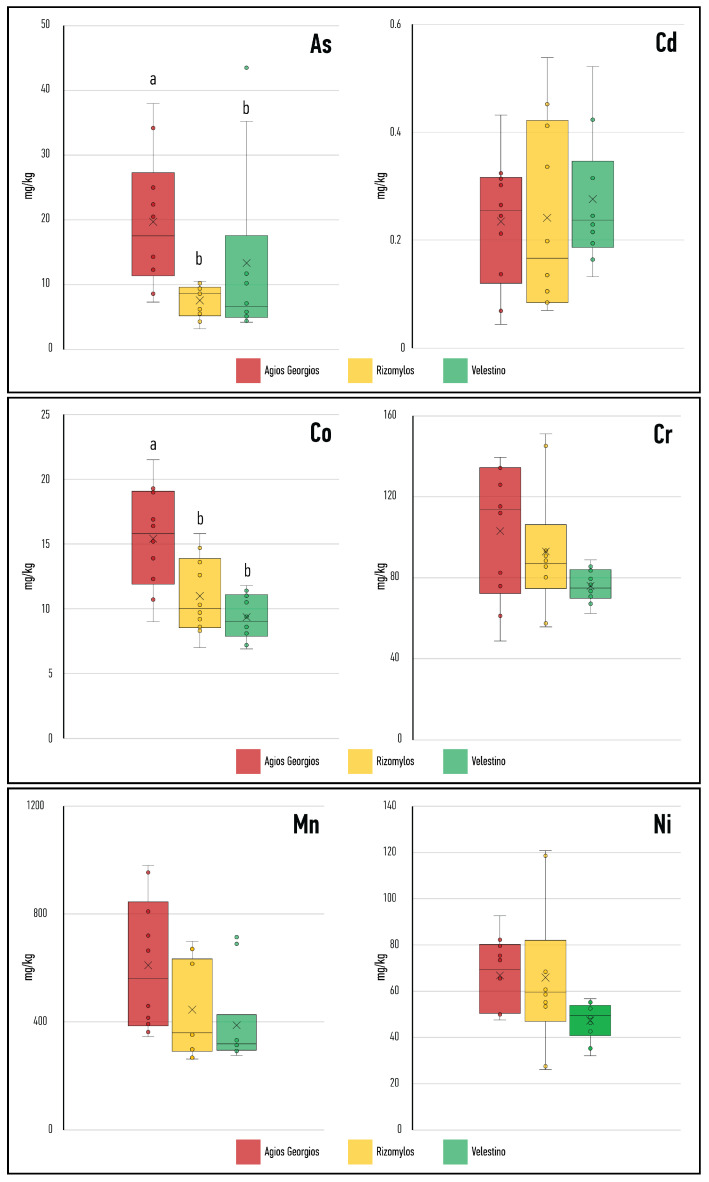

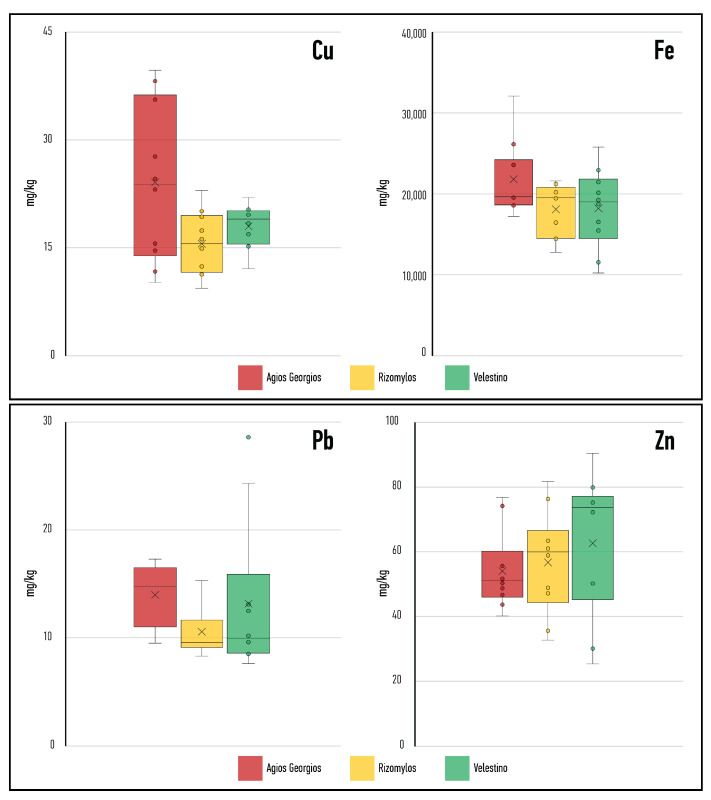
Statistical comparison between the PTE concentrations in the three areas; each area within the same sub-figure is depicted with a different color. Different letters denote statistically significant differences between groups AG, VL and/or RIZ (Tukey HSD test after one-way or Welch’s ANOVA). Where no letters are shown, ANOVA was performed, but it was not significant between groups (*p* > 0.05).

A statistical comparison between the PTE concentrations in the three areas was also performed, and the results are shown in Figure 4. The comparison attempted to find differences in the degree of pollution between the three areas (each area was represented by its ten sampling sites) for each PTE measured here.

A one-way ANOVA was performed for the sites when homogeneity of variances was satisfied; in the opposite case, Welch’s ANOVA was performed. For most PTEs, there were no significant differences between sites, and this may be partly explained by the great variability within the sites (AG, VL and RIZ), with some hotspots of higher pollution and some less polluted spots, as inferred from Table 2. For Co concentrations, however, the differences were significant (one-way ANOVA, F = 10.567, *p* < 0.001) with AG being more polluted than VL or RIZ. These results could not be confounded by the intra-variability within the area. The same was true for As, with significant differences (Welch’s ANOVA, F = 6.750, *p* < 0.05), with AG being the most polluted, VL intermediate and RIZ being the least polluted area.

### 3.3. Soil Contamination Assessment Indices

Calculations of the CF of the PTEs and the PLI of each sample classification in the three urban areas are illustrated in Table 2. Among the measured contents of PTEs, 152 values indicated a low degree of contamination (CF < 1), 108 values showed a moderate degree of contamination (1 ≤ CF < 3), 9 significant contamination (3 ≤ CF < 6) and 1 very high contamination (CF ≥ 6). In AG, 50% of the samples were of low contamination, 43% of moderate and 7% of considerable contamination. Furthermore, 50% of the samples for As belonged to class III (considerable contamination). In VL, 58% of the samples were of low contamination, 40% of moderate, 1% of considerable and 1% of very high contamination. Lastly, in the RIZ area, 61% of the measured samples scored low in contamination, while 37% showed moderate and 2% showed considerable contamination.

**Table 2:** CF and PLI of the potentially toxic elements in the three urban areas.

	No. of Samples	Cr	Mn	Co	Ni	Cu	Zn	As	Cd	Pb	PLI
		mg kg^−1^									
Soil (AG)	1AG	2.26	1.09	1.50	2.26	0.71	0.74	2.13	0.11	0.60	0.946
	2AG	2.27	1.18	1.68	2.53	0.92	0.79	3.00	0.17	0.64	1.116
	3AG	2.35	1.61	1.90	2.74	1.02	1.10	5.56	0.60	0.61	1.524
	4AG	2.12	1.56	1.71	2.60	0.98	1.06	5.01	0.52	0.56	1.412
	5AG	1.88	1.33	1.34	3.19	0.63	0.75	2.10	0.33	0.63	1.094
	6AG	1.94	0.75	1.45	2.84	0.59	0.72	1.80	0.65	0.53	1.052
	7AG	1.39	0.64	1.09	1.72	0.38	0.67	3.65	0.79	0.35	0.909
	8AG	1.28	0.68	1.23	1.64	0.40	0.70	3.28	0.74	0.41	0.924
	9AG	0.82	0.59	0.80	1.77	0.30	0.62	1.07	1.05	0.38	0.726
	10AG	0.86	0.57	0.95	1.74	0.26	0.57	1.26	0.77	0.46	0.722
	**Average**	**1.72**	**1.00**	**1.36**	**2.30**	**0.62**	**0.77**	**2.89**	**0.57**	**0.52**	**1.092**
Soil (VL)	1VL	1.49	0.67	0.61	1.91	0.51	1.14	0.64	1.27	0.46	0.861
	2VL	1.44	0.65	0.64	1.73	0.52	1.09	0.75	1.03	0.49	0.845
	3VL	1.05	1.49	1.05	1.11	0.57	1.08	6.37	0.47	1.06	1.150
	4VL	1.23	1.44	1.01	1.22	0.47	1.29	5.15	0.52	0.90	1.139
	5VL	1.41	0.61	0.71	1.60	0.31	0.43	0.84	0.40	0.28	0.614
	6VL	1.34	0.57	0.83	1.47	0.40	0.36	0.91	0.32	0.32	0.616
	7VL	1.29	0.62	0.98	1.96	0.52	1.07	0.61	0.56	0.36	0.778
	8VL	1.20	0.63	0.93	1.84	0.50	1.03	1.04	0.78	0.38	0.840
	9VL	1.19	0.71	0.77	1.68	0.43	0.72	1.72	0.77	0.31	0.804
	10VL	1.13	0.69	0.76	1.81	0.39	0.73	1.49	0.60	0.32	0.764
	**Average**	**1.28**	**0.81**	**0.83**	**1.63**	**0.46**	**0.89**	**1.95**	**0.67**	**0.49**	**0.896**
Soil (RIZ)	1RIZ	1.49	1.46	1.20	2.41	0.45	0.70	1.37	1.31	0.57	1.080
	2RΙΖ	1.57	1.40	1.12	2.36	0.50	0.67	1.26	1.10	0.54	1.041
	3RΙΖ	2.54	1.30	1.40	4.17	0.59	1.17	0.80	0.20	0.40	0.983
	4RΙΖ	2.44	1.28	1.30	4.09	0.52	1.09	0.91	0.26	0.38	0.979
	5RΙΖ	0.94	0.56	0.73	0.95	0.24	0.47	1.27	0.17	0.36	0.529
	6RΙΖ	0.97	0.55	0.81	0.90	0.29	0.51	1.33	0.20	0.35	0.561
	7RΙΖ	1.53	0.76	0.86	1.90	0.38	0.87	1.49	0.82	0.34	0.863
	8RΙΖ	1.44	0.73	0.91	1.84	0.42	0.91	1.54	1.00	0.33	0.887
	9RΙΖ	1.35	0.62	0.62	2.09	0.32	0.88	0.47	0.33	0.31	0.625
	10RΙΖ	1.38	0.63	0.76	2.02	0.30	0.84	0.63	0.48	0.35	0.691
	**Average**	**1.56**	**0.93**	**0.97**	**2.27**	**0.40**	**0.81**	**1.11**	**0.59**	**0.39**	**0.863**
**Legend**											
CF	Class I CF < 1		Class II 1 ≤ CF < 3			Class III 3 ≤ CF < 6			Class IV CF ≥ 6		
PLI	Index I PLI < 0.7		Index II 0.7 < PLI < 1		Index III 1 < PLI < 2		Index IV 2 < PLI < 3		Index V PLI > 3		

The PLIs for the three areas studied ranged from 0.561 to 1.524. Among the 30 samples, 11 fell under Index III (moderately polluted), 13 under Index II (slightly polluted) and 6 under Index I (unpolluted). The highest number of samples classified as moderately polluted were collected in urban areas of Agios Georgios, indicating that AG is the most polluted area (Table S7). Furthermore, none of the samples from AG are characterized as unpolluted. Nevertheless, calculation of the Igeo showed that in AG, 66% of the 90 measurements were uncontaminated, 27% were slightly contaminated and 7% moderately contaminated. In the case of Ni, 90% of the samples belonged to class 1 (slightly contaminated) and 10% to class 2 (moderately contaminated), respectively. A total of 80% of the samples collected were slightly to moderately contaminated with As (Table S8). In AG, the most contaminated samples were collected from parks, and in RIZ, the one moderately contaminated soil sample originated from a school plot.

### 3.4. Dimension Reduction and Clustering of Results

**Figure 5:**
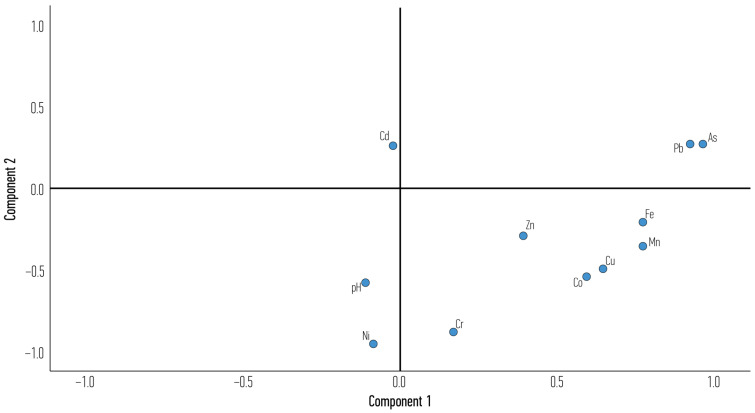
Component plot in rotated space.

The pseudo-total concentrations and the pH values of the 30 soil samples were subjected to a PCA with an Oblimin rotation. The data were deemed appropriate for this technique (correlation matrix determinant = 1.15 × 10^−5^; KMO = 0.676; Bartlett’s test *p* < 0.001). Based on the eigenvalues from the scree plot from the initial run, a forced two-factor solution was chosen. The first component of the two-factor solution explained 49.64% of the variance, while the second component explained an additional 17.43% (cumulative percentage of 67.07%). Only high loadings on each factor (≥0.5) were obtained. The results (Table 3 and Figure 5) show strong positive loadings for As, Pb, Mn, Fe, Cu and Co on the first component while Ni, Cr, Co and the pH group loaded negatively along the second component. On the contrary, Zn and Cd did not load substantially on any column (they exhibited loadings of less than 0.5).

**Figure 6:**
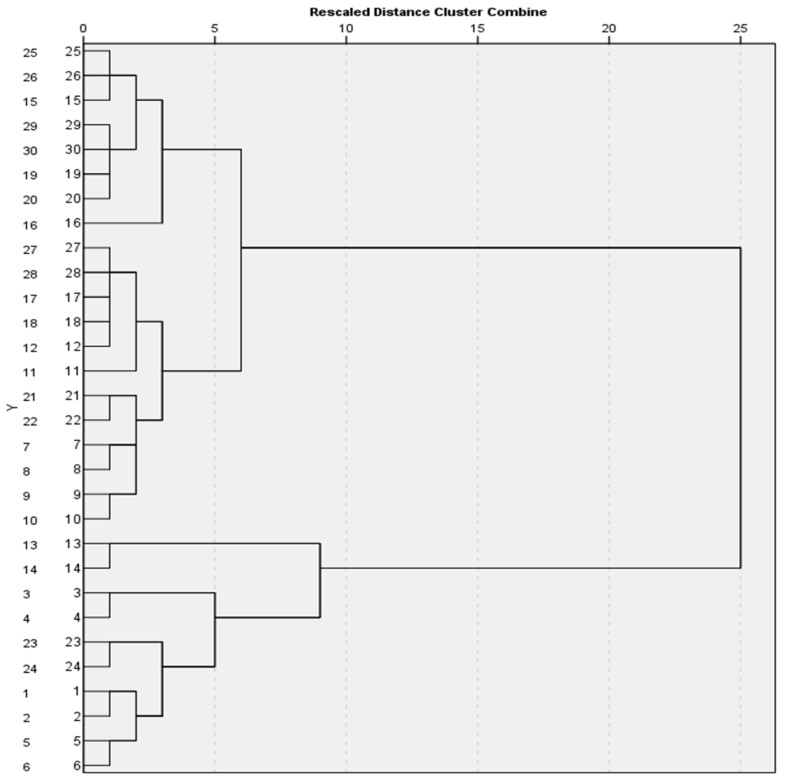
Hierarchical dendrogram of measured PTEs. Key: codes 1 to 10 belong to AG, 11 to 20 to VL and 21 to 30 to RIZ.

Hierarchical cluster analysis (Ward’s method utilizing squared Euclidian distance) on the transformed data (z-scores) revealed two distinct clusters, as shown in Figure 6.

The subsequent K-means Cluster Analysis created two distinct groups; the first comprised 21 cases and the second 9 cases. All parameters except pH value and Cd concentration differentiated between the two clusters (Table 4), with the most prominent differences being between the Mn values, Fe values, Co values and Cu values. Group 1 showed on average lower concentrations of PTEs than Group 2. Group 2 comprised four sites from AG, three from VL and two from RIZ.

## 4. Discussion

The current research evaluated 10 PTEs in soils from three urban areas surrounding the steel industrial zone of Volos (Greece). An assessment of the PTEs using CF, PLI and Igeo was conducted. Finally, the PTEs were grouped together due to possible common sources, while there was also a clear division of the soils into two groups.

At a quick glance, AG is the most polluted area of the three, being in direct vicinity of the steel plant; it has also been the area of interest for previous research on Volos for the same reason [47]. In that study, however [47], the points examined were cultivated fields in AG close to the industry. Those data (year 2017) showed similar average values to ours (only AG data) for Zn, Mn, Fe and Cu, and higher values for Cd, Co, Cr and As, but lower for Ni and Pb. The main industrial area (Industrial Park B), placed southeast of the steel plant [48] showed similar but slightly elevated values for Zn, higher values than ours for Cu and Cd, but again lower than ours for Ni, according to [49]. Another sampling campaign in 2019 [48] within the park verified high values for Zn and Cd and values similar to ours (AG) for As, Pb, Fe and Ni. In that campaign, VL points closest to the steel plant showed values higher than ours for Zn, Cd, Mn, Fe, Ni, Cu and Pb but similar for As [48]. These comparisons are only indicative because the land uses in the previous studies [47,48,49] were focused on peri-urban (agricultural and industrial) activities, while ours were from clear urban settings; what they show is that PTE enrichment in the industrial soils possibly has a carry-over effect to the near urban settlements. Another meaningful comparison may be with the urban agglomeration of Volos, the prefectural unit capital, near Industrial Park B, which, however, is additionally burdened by traffic, port activities and a cement factory on its east side [50,51]. In this context, when comparing the urban soils in AG, VL and RIZ with a series of historical data (2019–2020) for Volos, it can be noted that Cr, Mn and Ni concentrations were almost identical to ours, while other PTEs (Co, Cu, Zn, Cd and Pb) were considerably higher [52]. This means that we may attribute the presence of these PTEs to the steel industry to a high extent, or, in the case of Ni, to the site’s geochemical composition also [52]. On the other hand, Cu, Zn, Cd and Pb particles may primarily arise from coal burning for heating and from the wear and tear of automobiles [53] within the city, which is among the 10 largest in Greece. Additional sources may include wirings, pigments and plastics, as well as e-waste [54].

In any case, it was also of interest to compare found values to the Canadian SQGs. These guidelines have been set as a part of a stepwise process in risk assessment so that soils are protected from the harmful effects of toxic constituents; action is taken when levels are above certain limits. It is also noted that these limits are correlated to land uses; for example, there are different limits for residential areas in relation to agricultural fields. When compared to residential SQG values, the majority of Cr and Ni levels were above the limits in all three sites, while this was also true for AG and As concentrations. Exceedances of other set limits (see Table 1) were also found for many PTEs on a case-by-case basis. Contamination of soil and edible plants with Cr generates substantial risk for human populations, including cancer. Ni contamination also poses serious environmental concern when entering food production systems [55]; it negatively affects human physiology, and, except for metallic Ni, it may cause cancer [56]. Finally, As is an extremely toxic metalloid that elicits diseases ranging from dermal hyperkeratosis to diabetes, neurodegeneration and certain forms of cancer [57]. As such, these elevated concentrations raise concerns, and they should be frequently measured.

The findings were further supported by the calculation of soil contamination indices, such as CF. These indices provide valuable insights into the extent of metal contamination in the areas studied, particularly in relation to industrial activities. In the case of AG, the CF values indicated moderate contamination for PTEs, including Cr, Mn, Co, Ni and As. The presence of these elements at elevated concentrations in AG suggests a direct influence from industrial sources, most notably the nearby steel manufacturing facility [31,47]. In two other areas, VL and RIZ, the soil contamination patterns were slightly different, yet still noteworthy. The CF values in these two regions classified the soils as moderately contaminated with Cr, Ni and As. The elevated CF values for Cr in AG were anticipated, especially in soil samples collected from urban locations within close proximity to the steel factory. Previous studies have documented similar trends, linking Cr contamination to industrial activities. Moreover, during industrial processes such as smelting and refining, Ni, Cr and Mn may be emitted into the atmosphere. Additionally, Ni is released from coal and combustion as well as steel manufacturing [58,59]. The classification of CF values for Cd, Cu and Pb in this study aligns with the findings of Antoniadis et al. [57], confirming that similar contamination patterns have been observed in other regions with comparable industrial influences. Additionally, the CF values for Co and Mn in the AG area fall within the same category of moderate contamination, as reported by Antoniadis et al. [60]. These findings highlight a level of consistency in soil pollution trends, emphasizing the impact of industrial emissions on soil quality across different locations [10,61,62].

PLI values were determined for the three areas studied, with measurements indicating a value of 1.092 for AG, 0.896 for VL and 0.863 for RIZ. Among these, the highest PLI values were recorded in AG and VL, which can likely be attributed to the relative proximity of these urban areas to the steel factory. This industrial site is located approximately 6.5 km from AG and 7 km from VL, suggesting that emissions and forms of pollution associated with steel production have contributed to the contamination levels observed in these regions [63]. Furthermore, comparative studies highlight the context of pollution levels in industrial areas. Qing et al. [31] reported significantly higher PLI values in the steel industrial city of Anshan in China, emphasizing the substantial environmental burden imposed by large-scale industrial activities. The elevated contamination levels in that region serve as an example of how prolonged exposure to emissions from steel factories can lead to severe pollution. Our findings are also in alignment with the average PLI values reported by Antoniadis et al. [60], which further reinforce the reliability and consistency of our data when compared with previous studies. The similarity in reported values suggests that the levels of contamination in the studied regions fall within a predictable range for areas affected by industrial pollution. Similarly, research conducted by Doležalová Weissmannová et al. [64] assessed the total PLI values in the Ostrava region of the Czech Republic and classified it as moderately polluted. These comparisons indicate that while the pollution levels in AG and VL are notable, they are not unprecedented in the context of regions impacted by industrial activities. The findings underscore the importance of continued monitoring and mitigation strategies to manage contamination and minimize its environmental and public health effects.

In the urban area of AG, the calculated Igeo values indicate that the soils exhibit slight contamination with Cr, Ni and As. A particularly noteworthy observation is that, in the case of As, 50% of the analyzed soil samples were classified within the category of moderate contamination, suggesting a more pronounced presence of this element in the area. While the average Igeo values for Cr were found to be lower than those reported in previous studies, the corresponding values for Ni were comparatively higher. Such findings highlight the varying degrees of metal accumulation in urban soils and emphasize the need for localized assessments to better understand contamination patterns [10,30]. Additionally, a study conducted by Chen et al. [65] examined urban soil contamination in the Kundulun District in Baotou (Mongolia), a region recognized for its steel manufacturing industry. Their assessment, which also utilized the Igeo index, revealed lower concentrations of Cr, Ni and As in comparison to our findings. This suggests that despite industrial activities in both areas, the levels of contamination observed in AG are relatively elevated, warranting further investigation into potential pollution sources and their environmental impact.

The “trend” of AG being the most polluted area and RIZ being the least polluted in our study is statistically corroborated by two PTEs only, namely Co and As. The lack of differences for the other PTEs may be attributed to the large standard deviations within each settlement. As stated in the results section, in all three areas there were some hotspots of high contamination, accompanied by other spots of lower contamination. This intra-variability does not allow for a clear depiction of differences between the three selected settlements. In any case, the differences for Co and As were high enough, and AG was the most polluted for both PTEs, while RIZ was the least polluted. These differences cannot readily be explained, since all three settlements are small (533 inhabitants in AG, 2919 in VL and 1214 in RIZ) according to the 2021 national census [66] and the people mostly work in agriculture or industry, with RIZ being the furthest from the steel plant. The proximity of AG to the steel plant may be responsible for Co enrichment since this metal is used as an alternative to Ni in the sintering process and as a binder for carbides in hard metal production [67]. The differences in As contamination are also not directly explained since As is not intentionally used for any industrial processes taking place in Volos. It is, however, known that metal smelting and coal combustion contribute more than 60% of the anthropogenic enrichment of air with As [68], which can then precipitate on nearby soil. Furthermore, workers in steel production and steel quality control have exhibited elevated As levels in urine, which signifies some As exposure [69,70]. It is therefore possible that these differences are connected to the distance from the steel plant, with AG being the closest and RIZ being the furthest. “Hotspots” have also been revealed; for example, in one site in VL for As. As redistribution due to natural geological processes also cannot be ruled out [71]. It should likewise be noted that both non-carcinogenic and carcinogenic risk from PTEs for these residents is additive; this means that high levels of less hazardous PTEs (such as Zn, Cu and Fe) also increase the risk [72] when accompanying modest or even low levels of more toxic PTEs simultaneously.

Grouping of PTEs through a PCA can reveal some interesting intercalations [73]. It looks as if most PTEs used in heavy industry activities (Mn, Fe, Cu, Co and Pb), as well as PTEs that manifest themselves in these environments (As, [69]) co-exist together (first component that explains 49.64% of the variance within the examined system). At the same time, additional variation is explained by a second component characterized by the absence (lower than average values) of Ni, Co and Cr in soils, which are also less alkaline than the average of the examined soils (average pH = 8.09). No causative explanation has been considered for low PTEs and lower pH here; it is known that mobility of most metals is higher in acidic soils [37,74]. As such, pollution is more dangerous at low pH; however, pseudo-total concentrations were measured here as no such data are available for the metal phase and speciation. It is possible that these soils are the least impacted by alloy production in the general urban vicinity of the industrial park. Finally, cluster analysis verified the presence of two clusters: one is characterized by higher anthropogenic impact in the form of higher values of Fe, Mn, Co and Cu, while the rest of the sites (21 in total) belong to the other cluster of lower ambient PTE concentrations, especially in the case of Fe, Mn, Co and Cu. As expected, the high-impact cluster contained most sites from AG, fewer from VL and the fewest from RIZ. A similar analysis based on PTE enrichment also distinguished sites according to their (vicinity to) various land uses [75].

Taken as a whole, the above-mentioned results show various degrees of the contamination of the examined area with PTEs, with significant spatial differences which may also be species-specific. Limitations of the study may include the following: A limited number of samples per area and a lack of time series (the sampling was performed only once during the summer of 2022). Furthermore, no historical data for the exact same areas are available, but comparisons can be made with extensive campaigns in agricultural and industrial fields in Volos, which is a city impacted by many environmental stressors. For the same reason (the presence of touristic/industrial/port activities, traffic congestion and the existence of both industrial and municipal wastes), the pollution cannot be attributable to a handful of reasons only. Nevertheless, correlations and co-presences of PTEs can emerge from the present set of data.

The results shown here, together with similar research on inorganic soil pollution in the extended area, reveal a situation worthy of attention: the urban agglomeration of Volos and the surrounding settlements are touristic destinations and lively cities; however, they are at risk from past and current industrial activities. As such, pollution monitoring is vital for the area and should be duly repeated.

## 5. Conclusions

Soil samples from parks, playgrounds and roadsides were collected from three urban settlements near the industrial area of Volos city, Central Greece, and PTE concentrations were assessed. The pseudo-total concentrations of Cr, Ni and As were elevated compared to worldwide background levels for many of the samples. There was also an exceedance of Canadian residential SQGs for Cr and Ni and an exceedance of the Dutch Target Values, especially for Ni. Comparisons between the three sites showed that AG was significantly more polluted than the other two for Co. AG was also the most polluted for As, and VL was intermediate and RIZ was the least polluted area for As. When calculating well-known pollution indices, CF revealed moderate, considerable or even very high contamination for Cr, Mn, Co, Ni or As in a few samples. Calculation of PLI showed moderate pollution for AG and slight pollution for the other two sites, while Igeo was mostly elevated for AG, Ni or As. The PCA showed a co-existence of PTEs used in heavy industry activities (Mn, Fe, Cu, Co and Pb) and of As, while there was also a second component characterized by the absence (lower than average values) of Ni, Co and Cr in less alkaline soils. Finally, it was evident that soil samples belonged to two distinct clusters: one was anthropogenically impacted with higher values of Fe, Mn, Co and Cu, while the rest (21 sites in total) belonged to the other cluster of lower ambient PTE concentrations. The area examined is possibly affected by the steel-making industrial unit and the industrial park (second industrial zone) of the city, and it should be frequently monitored.

## Figures and Tables

**Table 1:** Comparison of measured concentrations with national and international guidelines.

	No. of Samples	Cr	Mn	Fe	Co	Ni	Cu	Zn	As	Cd	Pb
			mg kg^−1^								
Soil (AG)	1AG	*#●			#●	*#●			*●		
	2AG	*#●			#●	*#●			*●		
	3AG	*#●	●		#●	*#●	#●	●	*#●		
	4AG	*#●	●		#●	*#●	#	●	*#●		
	5AG	*#●			#●	*#●			*●		
	6AG	*#●			#●	*#●			*●		
	7AG	*●			#●	*#●			*●		
	8AG	*●			#●	*#●			*●		
	9AG					*#●			●	●	
	10AG					*#●			●		
Soil (VL)	1VL	*●				*#●		●		●	
	2VL	*●				*#●		●		●	
	3VL	●			#●	●		●	*#●		●
	4VL	*●			#●	#●		●	*#●		●
	5VL	*●				*#●					
	6VL	*●				#●					
	7VL	*●				*#●		●			
	8VL	*●				*#●		●	●		
	9VL	*●				*#●			●		
	10VL	*●				*#●			●		
Soil (RIZ)	1RIZ	*●			#●	*#●			●	●	
	2RΙΖ	*●			#●	*#●			●	●	
	3RΙΖ	*#●			#●	*#●		●			
	4RΙΖ	*#●			#●	*#●		●			
	5RΙΖ										
	6RΙΖ								●		
	7RΙΖ	*●				*#●			●		
	8RΙΖ	*●				*#●			●	●	
	9RΙΖ	*●				*#●					
	10RΙΖ	*●				*#●					

Key: Gray cells above the limits in Ministerial Decree DDA/41828/630/2023 [39]; * above the Canadian SQGs [40]; # above the Dutch Target Values; ● above the background values in Kabata-Pendias [33] (for Mn and Fe above the background values in [42]).

**Table 3:** Rotated component matrix from PCA.

Parameter	Component 1	Component 2
As	0.966	
Pb	0.930	
Mn	0.777	
Fe	0.778	
Cu	0.647	
Co	0.595	−0.541
Zn		
Ni		−0.950
Cr		−0.887
pH		−0.573
Cd		

**Table 4:** ANOVA comparisons between established groups (1 and 2) from K-means Cluster Analysis.

Parameter	F Value	*p* Value
Cr	4.842	0.036
Mn	15.992	<0.01
Fe	30.962	<0.01
Co	13.641	<0.01
Ni	4.577	0.041
Cu	19.822	<0.01
Zn	5.351	0.028
As	4.353	0.046
Cd	0.416	0.524
Pb	5.922	0.022
pH	0.119	0.733

## Data Availability

The original contributions presented in this study are included in the article/Supplementary Material. Further inquiries can be directed to the corresponding author.

## Supplementary Materials

The following supporting information can be downloaded at https://www.mdpi.com/article/10.3390/toxics13050351/s1. Table S1: ICP-MS operating conditions and acquisition parameters. Table S2: LOD/LOQ for the examined PTEs. Table S3: The geographical position of soil samples. Table S4: Classification categories of Pollution Load Index (PLI) [1]. Table S5: Classification categories of Geo-accumulation Index (Igeo) [2]. Table S6: A Pseudo—total concentrations of the 10 studied potentially toxic elements (mg kg^−1^) (mean± SD) in the three urban areas. B Some internationally accepted limits used for comparison in main text. Table S7: Pollution Load Index (PLI) for the three urban areas. Table S8: Igeo index values for the three urban areas.
