# Supplementary material for: Evaluation of Potential Toxic Elements in Soils from Three Urban Areas Surrounding a Steel Industrial Zone
Source: Toxics. 2025 Apr 28;13(5):351. doi: 10.3390/toxics13050351 (PMC12116155; doi:10.3390/toxics13050351)
Supplement: Supplementary file 1 [file toxics-13-00351-s001.zip › toxics-3556161-supplementary.pdf]

SUPPLEMENTARY MATERIAL

Table S1. ICP-MS operating conditions and acquisition parameters

Operating conditions	Values
Instrument	Agilent 7700
Cone	Ni
Auxiliary Gas Flow	0.9 L/min
Nebulizer Gas Flow	15 L/min
Peristaltic Pump Flow	0.1 L/min
He flow	4.2 mL/min
RF power	1550 W

Table S2. LOD/LOQ for the examined PTEs

Element	LOD μg/Kg	LOQ μg/kg
^{52}Cr	17.1	56.943
^{60}Ni	13.6	45.288
^{56}Fe	42	139.86
^{55}Mn	5.9	19.647
^{66}Zn	33.7	112.221
^{63}Cu	42.4	141.192
^{111}Cd	17.6	58.608
^{208}Pb	13.9	46.287
^{75}As	3.4	11.322
^{59}Co	9.8	32.634

Table S3. The geographical position of soil samples.

Soil sample ID	Location	Longitude	Latitute
Agios Georgios area			
1AG	Church	39°22'02''	22°46'42''
2AG	Church	39°22'01''	22°46'41''
3AG	Park	39°22'06''	22°46'39''

4AG	Park	39°22'07"	22°46'39"
5AG	Park	39°22'02"	22°46'38"
6AG	Park	39°22'04"	22°46'37"
7AG	Park	39°22'16"N	22°46'37"
8AG	Park	39°22'14"	22°46'38"
9AG	Central Square	39°22'17"	22°46'34"
10AG	Central Square	39°22'18"	22°46'35"
Velestino area			
1VL	Church	39°22'53"	22°44'59"
2VL	Church	39°22'56"	22°44'40"
3VL	School	39°22'41"	22°44'40"
4VL	Park	39°23'05"	22°44'48"
5VL	Playground	39°22'47"	22°44'42"
6VL	Park	39°23'01"	22°44'34"
7VL	Park	39°22'49"	22°44'31"
8VL	Park	39°22'46"	22°44'37"
9VL	School	39°22'49"	22°44'24"
10VL	Medical center	39°22'41"	22°44'20"
Rizomilos area			
1RIZ	School	39°25'36"	22°44'53"
2RIZ	Church	39°25'38"	22°44'58"
3RIZ	Church	39°25'32"	22°45'04"
4RIZ	Park	39°25'34"	22°45'05"
5RIZ	House	39°25'34"	22°44'43"
6RIZ	House	39°25'51"	22°44'45"
7RIZ	Square	39°25'45"	22°44'46"
8RIZ	Square	39°25'45"	22°44'48"
9RIZ	Road side	39°25'38"	22°45'06"
10RIZ	Road side	39°25'35"	22°44'59"

Table S4. Classification categories of Pollution Load Index (PLI) [1].

Indices	Values	Characterization
I	<0.7	unpolluted
II	0.7 - 1	slightly polluted
III	1 - 2	moderately polluted
IV	2 - 3	severely polluted
V	>3	heavily polluted

Table S5. Classification categories of Geo-accumulation Index (Igeo) [2].

Grades	Igeo Value	Characterization
0	≤ 0	Uncontaminated
1	0<Igeo≤1	Slightly contaminated
2	1<Igeo≤2	Moderately contaminated
3	2<Igeo≤3	From moderately contaminated to strongly contaminated
4	3<Igeo≤4	Strongly contaminated
5	4<Igeo≤5	Seriously contaminated
6	5<Igeo≤10	Extremely seriously contaminated

Table S6. A Pseudo – total concentrations of the 10 studied potentially toxic elements (mg kg^{-1}) (mean± SD) in the three urban areas. B Some internationally accepted limits used for comparison in main text.

	**No of Samples**	pH	Cr	Mn	Fe	Co	Ni	Cu	Zn	As	Cd	Pb
				mg kg^{-1}								
**Soil (Agios Georgios)**	1AG	8.01	134.3±0.80	664.3±0.45	19532.6±0.51	16.9±0.25	65.5±0.23	27.7±0.26	51.7±0.30	14.6±0.45	0.044±0.003	16.3±0.45
	2AG	8.12	135.3±0.56	720.1±0.40	23564.2±0.35	19.0±0.55	73.4±0.42	35.6±0.30	55.6±0.30	20.5±0.56	0.069±0.004	17.3±0.25
	3AG	8.25	139.6±0.55	980.3±0.49	32111.2±0.45	21.5±0.75	79.6±0.35	39.7±0.25	76.8±0.30	38.0±0.31	0.245±0.003	16.3±0.37
	4AG	8.33	126.0±0.35	954.2±0.35	26123.4±0.45	19.3±0.55	75.4±0.30	38.2±0.32	74.2±0.30	34.2±0.35	0.212±0.001	15.2±0.45
	5AG	8.33	112.0±0.25	809.1±0.45	23580.7±0.60	15.2±0.55	92.6±0.30	24.6±0.30	52.3±0.37	14.3±0.41	0.137±0.004	17.1±0.41
	6AG	8.23	115.3±0.55	459.2±0.45	19785.1±0.36	16.4±0.61	82.3±0.20	23.1±0.30	50.3±0.45	12.3±0.46	0.265±0.006	14.3±0.55
	7AG	8.16	82.5±0.40	392.0±0.35	18634.6±0.35	12.3±0.40	49.9±0.25	14.6±0.35	46.7±0.45	25.0±0.35	0.324±0.005	9.5±0.61
	8AG	8.21	75.9±0.30	415.2±0.45	17236.2±0.35	13.9±0.40	47.6±0.25	15.6±0.45	48.7±0.40	22.4±0.55	0.302±0.004	11.2±0.35
	9AG	7.99	48.7±0.32	362.6±0.45	18846.5±0.35	9.0±0.35	51.3±0.35	11.7±0.35	43.7±0.40	7.3±0.55	0.432±0.005	10.4±0.55
	10AG	8.02	51.4±0.42	345.6±0.85	18562.3±0.55	10.7±0.42	50.6±0.55	10.2±0.40	40.2±0.45	8.6±0.36	0.314±0.002	12.3±0.45
	**Maximum**	8.33	139.6	954.2	32111.2	21.5	92.6	39.7	76.8	38.0	0.432	17.3
	**Minimum**	7.99	48.7	345.6	19532.6	9.0	47.6	10.2	40.2	7.3	0.044	9.5
	**Average**	**8.17**	**102.1**	**610.3**	**21797.7**	**15.4**	**66.8**	**24.1**	**54.0**	**19.7**	**0.234**	**14.0**
**Soil (Velestino)**	1VL	8.13	88.8±0.35	322.8±0.35	10229.2±0.35	6.9±0.36	55.2±0.50	19.7±0.35	79.9±0.40	4.4±0.40	0.522±0.004	12.5±0.48
	2VL	8.23	85.6±0.30	314.2±0.40	19235.1±0.35	7.2±0.31	50.2±0.31	20.1±0.40	76.3±0.40	5.1±0.31	0.423±0.004	13.1±0.40
	3VL	7.86	62.4±0.30	714.2±0.40	25780.7±0.20	11.8±0.40	32.1±0.40	22.0±0.35	75.3±0.45	43.5±0.51	0.194±0.003	28.6±0.77
	4VL	8.23	73.4±0.30	689.2±0.40	20123.2±0.35	11.4±0.25	35.3±0.25	18.4±0.50	90.5±0.61	35.2±0.45	0.215±0.004	24.3±0.40

	5VL	8.07	83.6±0.40	292.2±0.45	11540.2±0.30	8.1±0.35	46.3±0.35	12.1±0.30	30.1±0.41	5.8±0.35	0.164±0.006	7.6±0.35
	6VL	7.66	79.5±0.35	275.4±0.60	15452.3±0.45	9.4±0.25	42.6±0.25	15.6±0.45	25.4±0.40	6.2±0.40	0.132±0.003	8.6±0.32
	7VL	8.21	76.6±0.35	296.6±0.45	22932.8±0.40	11.0±0.40	56.7±0.40	20.3±0.46	75.2±0.40	4.2±0.40	0.229±0.004	9.6±0.36
	8VL	8.14	71.6±0.35	302.4±0.46	21456.5±0.56	10.5±0.35	53.4±0.35	19.6±0.40	72.2±0.35	7.1±0.35	0.321±0.004	10.2±0.35
	9VL	8.21	70.7±0.35	340.2±0.50	18744.5±0.35	8.7±0.20	48.8±0.20	16.9±0.40	50.2±0.45	11.7±0.46	0.315±0.005	8.5±0.40
	10VL	8.32	67.2±0.21	332.1±0.35	16523.2±0.45	8.6±0.20	52.5±0.20	15.2±0.55	51.2±0.45	10.2±0.45	0.245±0.004	8.7±0.31
	**Maximum**	8.32	88.8	714.2	25780.7	11.8	56.7	22.0	90.5	43.5	0.522	28.6
	**Minimum**	7.66	62.4	275.4	10229.2	6.9	32.1	12.1	25.4	4.2	0.132	7.6
	**Average**	**8.11**	**75.9**	**387.9**	**18201.8**	**9.4**	**47.3**	**18.0**	**62.6**	**13.3**	**0.276**	**13.2**
**Soil (Rizomilos)**	1RIZ	7.98	88.4±0.30	699.5±0.51	21598.2±0.41	13.6±0.45	69.9±0.40	17.4±0.40	48.9±0.65	9.4±0.55	0.538±0.001	15.3±0.27
	2RIZ	8.39	93.4±0.50	670.1±0.40	20212.1±0.40	12.6±0.50	68.4±0.57	19.3±0.60	47.2±0.35	8.6±0.48	0.452±0.002	14.5±0.56
	3RIZ	8.26	151.1±0.45	621.7±0.46	21218.9±0.41	15.8±0.45	120.8±0.60	23.0±0.51	81.8±0.56	5.5±0.50	0.084±0.005	10.7±0.56
	4RIZ	8.21	145.2±0.40	615.6±0.50	19456.3±0.45	14.7±0.55	118.6±0.50	20.1±0.46	76.3±0.70	6.2±0.55	0.105±0.004	10.2±0.40
	5RIZ	8.12	55.7±0.45	267.4±0.56	14432.4±0.56	8.3±0.40	27.5±0.47	9.4±0.40	32.7±0.66	8.7±0.45	0.070±0.005	9.7±0.40
	6RIZ	8.17	57.5±0.95	262.7±0.55	16457.2±0.40	9.2±0.45	26.1±0.30	11.3±0.55	35.6±0.55	9.1±0.40	0.084±0.006	9.4±0.55
	7RIZ	8.05	90.9±0.40	366.3±0.55	20680.1±0.45	9.7±0.46	55.2±0.45	14.9±0.65	61.0±0.40	10.2±0.70	0.336±0.005	9.2±0.41
	8RIZ	8.16	85.6±0.45	352.3±0.42	19562.3±0.56	10.3±0.46	53.4±0.55	16.2±0.45	63.4±0.65	10.5±0.60	0.412±0.004	8.9±0.55
	9RIZ	8.23	80.3±0.45	298.4±0.47	12761.0±0.50	7.0±0.40	60.7±0.76	12.4±0.71	61.6±0.65	3.2±0.65	0.135±0.006	8.3±0.46
	10RIZ	8.18	82.1±0.45	302.1±0.35	14521.6±0.40	8.6±0.40	58.6±0.56	11.7±0.70	58.9±0.65	4.3±0.60	0.198±0.005	9.4±0.51
	**Maximum**	8.39	151.1	699.5	21598.2	15.8	120.8	23.0	81.8	10.5	0.538	15.3
	**Minimum**	7.98	80.3	262.7	12761.0	7.0	26.1	9.4	32.7	3.2	0.070	8.3
	**Average**	**8.18**	**93.0**	**445.6**	**18090.0**	**11.0**	**65.9**	**15.6**	**56.7**	**7.6**	**0.241**	**10.6**
			Cr	Mn	Fe	Co	Ni	Cu	Zn	As	Cd	Pb
			mg kg^{-1}									
**Limit values**	Ministerial Decree [3] (for pH>7)	100	-	-	-	105	100	200	30	1.5	100	37
	Canadian SQGs [4] (for residential use)	64	-	-	50	45	63	250	12	10	140	38
	Dutch Target Values [5]	139.6	-	-	9	35	36	140	29	0.8	85	39
	Kabata-Pendias (2011) [6]	59.5	-	-	11.3	29	38.9	70	6.83	0.41	27	31
	Haynes (2016) [7]	-	950	5.63 x 10^4	-	-	-	-	-	-	-	40

Table S7. Pollution Load Index (PLI) for the three urban areas.

Area	PLI	Index
Agios Georgios	1.092	moderately polluted
Velestino	0.896	slightly polluted
Rizomilos	0.863	slightly polluted

Legend

**PLI**	**Index I**	**Index II**	**Index III**	**Index IV**	**Index V**
	**PLI < 0.7**	**0.7 < PLI < 1**	**1 < PLI < 2**	**2 < PLI < 3**	**PLI > 3**

Table S8. Igeo index values for the three urban areas.

Agios Georgios area									
Sample	$Igeo_{Cr}$	$Igeo_{Mn}$	$Igeo_{Co}$	$Igeo_{Ni}$	$Igeo_{Cu}$	$Igeo_{Zn}$	$Igeo_{As}$	$Igeo_{Cd}$	$Igeo_{Pb}$
1AG	0.5896	-0.1163	-0.0035	0.5902	-1.0727	-1.0215	0.5072	-3.8000	-1.3146
2AG	0.6002	0.0002	0.1647	0.7548	-0.7129	-0.9172	1.0007	-3.1559	-1.2271
3AG	0.6454	0.4453	0.3405	0.8717	-0.5563	-0.4504	1.8903	-1.3253	-1.3094
4AG	0.4975	0.4063	0.1873	0.7935	-0.6112	-0.5009	1.7391	-1.5365	-1.4139
5AG	0.3272	0.1684	-0.1615	1.0896	-1.2435	-1.0044	0.4858	-2.1674	-1.2406
6AG	0.3695	-0.6489	-0.0476	0.9199	-1.3368	-1.0618	0.2637	-1.2146	-1.5019
7AG	-0.1137	-0.8771	-0.4626	0.1977	-1.9966	-1.1681	1.2847	-0.9234	-2.0977
8AG	-0.2338	-0.7942	-0.2862	0.1299	-1.9032	-1.1084	1.1286	-1.0260	-1.8544
9AG	-0.8731	-0.9896	-0.9093	0.2372	-2.3181	-1.2654	-0.4814	-0.5103	-1.9655
10AG	-0.7961	-1.0589	-0.6653	0.2181	-2.5162	-1.3851	-0.2525	-0.9698	-1.7193
Average	**0.1013**	**-0.3465**	**-0.1843**	**0.5803**	**-1.4267**	**-0.9883**	**0.7566**	**-1.6629**	**-1.5644**
Velestino area									
Sample	$Igeo_{Cr}$	$Igeo_{Mn}$	$Igeo_{Co}$	$Igeo_{Ni}$	$Igeo_{Cu}$	$Igeo_{Zn}$	$Igeo_{As}$	$Igeo_{Cd}$	$Igeo_{Pb}$
1VL	-5.9014	-10.0642	-4.7846	-4.5131	-6.8473	-6.5235	-3.9958	1.0507	-6.4453
2VL	-5.9551	-10.1032	-4.7335	-4.6513	-6.8192	-6.5899	-3.7782	0.7464	-6.3832
3VL	-6.4118	-8.9186	-4.0165	-5.2944	-6.6871	-6.6084	-0.6865	-0.3779	-5.2593
4VL	-6.1769	-8.9700	-4.0705	-5.1593	-6.9467	-6.3437	-0.9912	-0.2299	-5.4919
5VL	-5.9892	-10.2078	-4.5722	-4.7692	-7.5512	-7.9299	-3.5998	-0.6225	-7.1707
6VL	-6.0617	-10.2934	-4.3488	-4.8881	-7.1849	-8.1768	-3.4965	-0.9337	-6.9904
7VL	-6.1158	-10.1863	-4.1194	-4.4749	-6.8048	-6.6104	-4.0622	-0.1363	-6.8277

8VL	-6.2127	-10.1584	-4.1891	-4.5622	-6.8556	-6.6696	-3.3009	0.3483	-6.7442
9VL	-6.2302	-9.9884	-4.4671	-4.6920	-7.0688	-7.1943	-2.5759	0.3188	-7.0151
10VL	-6.3042	-10.0233	-4.4771	-4.5867	-7.2224	-7.1655	-2.7782	-0.0415	-6.9737
Average	**-6.1359**	**-9.8914**	**-4.3779**	**-4.7591**	**-6.9988**	**-6.9812**	**-2.9265**	**0.0122**	**-6.5301**

Rizomilos area

Sample	$Igeo_{Cr}$	$Igeo_{Mn}$	$Igeo_{Co}$	$Igeo_{Ni}$	$Igeo_{Cu}$	$Igeo_{Zn}$	$Igeo_{As}$	$Igeo_{Cd}$	$Igeo_{Pb}$
1RIZ	-5.9088	-8.9485	-3.8204	-4.1729	-7.0235	-7.2305	-2.8990	1.0932	-6.1626
2RIZ	-5.8292	-9.0105	-3.9261	-4.2050	-6.8778	-7.2828	-3.0244	0.8420	-6.2368
3RIZ	-5.1352	-9.1186	-3.5986	-3.3842	-6.6260	-6.4893	-3.6708	-1.5896	-6.6781
4RIZ	-5.1927	-9.1329	-3.7037	-3.4110	-6.8192	-6.5899	-3.4965	-1.2639	-6.7442
5RIZ	-6.5762	-10.3360	-4.5287	-5.5222	-7.9161	-7.8125	-3.0098	-1.8431	-6.8153
6RIZ	-6.5291	-10.3615	-4.3798	-5.5949	-7.6501	-7.6897	-2.9429	-1.5858	-6.8621
7RIZ	-5.8687	-9.8819	-4.3061	-4.5155	-7.2475	-6.9134	-2.7771	0.4142	-6.8869
8RIZ	-5.9551	-9.9381	-4.2169	-4.5622	-7.1304	-6.8571	-2.7364	0.7084	-6.9409
9RIZ	-6.0478	-10.1775	-4.7710	-4.3777	-7.5119	-6.8996	-4.4538	-0.8995	-7.0471
10RIZ	-6.0153	-10.1599	-4.4771	-4.4281	-7.5999	-6.9633	-4.0244	-0.3488	-6.8621
Average	**-5.9058**	**-9.7065**	**-4.1729**	**-4.4174**	**-7.2403**	**-7.0728**	**-3.3035**	**-0.4473**	**-6.7236**

Legend

**Igeo**	Class 0	Class 1	Class 2	Class 3	Class 4	Class 5	Class 6
	Igeo≤0	0<Igeo≤1	1<Igeo≤2	2<Igeo≤3	3<Igeo≤4	4<Igeo≤5	5<Igeo≤10

References

1. Tomlinson, D.; Wilson, J.; Harris, C.; Jeffrey, D. Problems in the assessment of heavy-metal levels in estuaries and the formation of a pollution index. *Helgoländer meeresuntersuchungen* **1980**, *33*, 566-575.
2. Müller, G. Index of geo-accumulation in sediments of the Rhine River. *GeoJournal* **1969**, *2*, 108-118.
3. Ministerial Decree DDA/41828/630/2023 og 21 April 2023 on the protection of the environment, and in particular of the soil, when sewage sludge is used in agriculture. **2023**, 27301 - 27332
4. Canadian Council of Ministers of the Environment Available online: https://ccme.ca/en/summary-table (accessed on 15 April 2025)
5. Bini, C. From soil contamination to land restoration. *Contaminated Soils: Environmental Impact, Disposal and Treatment* **2011**, 97-137.
6. Kabata-Pendias, E. *Trace Elements in Soils and Plants*, 4th ed.; CRC Press: Boca Raton: FL, USA, 2011.
7. Haynes, W.M. *CRC Handbook of Chemistry and Physics (97th ed.)*; CRC Press.: 2016.
